# Draft Genome Sequence of Rice Isolate *Pseudomonas chlororaphis* EA105

**DOI:** 10.1128/genomeA.01342-14

**Published:** 2014-12-24

**Authors:** Lucy M. McCully, Adam S. Bitzer, Carla A. Spence, Harsh P. Bais, Mark W. Silby

**Affiliations:** a Department of Biology, University of Massachusetts Dartmouth, North Dartmouth, Massachusetts, USA; b Department of Biological Sciences, University of Delaware, Newark, Delaware, USA; c Delaware Biotechnology Institute, Newark, Delaware, USA; d Department of Plant and Soil Sciences, University of Delaware, Newark, Delaware, USA

## Abstract

*Pseudomonas chlororaphis* EA105, a strain isolated from rice rhizosphere, has shown antagonistic activities against a rice fungal pathogen, and could be important in defense against rice blast. We report the draft genome sequence of EA105, which is an estimated size of 6.6 Mb.

## GENOME ANNOUNCEMENT

*Pseudomonas chlororaphis* strain EA105 was isolated from rice cultivar M-104 and is capable of inhibiting growth of *Magnaporthe oryzae*, the fungus responsible for rice blast, as well as reducing disease in the rice plant with pretreatment (1). *P. chlororaphis* have been studied as biocontrol agents in tomatoes (2), canola (3), and cocoyam roots (4); several strains exhibit antifungal activities to protect plants from pathogens (2, 4–6), while others induce systemic resistance in their host (7, 8). *Pseudomonas* species are well suited for use as biocontrol agents due to the range of secondary metabolites they produce (5, 9). Biocontrol provides a safer alternative to chemical pesticides for crop protection (10). Analysis of this genome will help provide insight into the mechanisms underlying biocontrol activity by EA105, including production of antifungal compounds, and systems by which EA105 interacts with pathogens and its plant host.

Genomic DNA was isolated from *P. chlororaphis* EA105 using the Wizard Genomic DNA purification kit (Promega). The genomic library was prepared using a Nextera XT sequencing library preparation kit (Illumina). Sequencing was carried out at Tufts University Genomics Core using a MiSeq genome sequencer (Illumina), which generated 3,188,442 2 × 250 bp paired end reads. The genome was assembled using CLC v7.5, producing 74 contigs ranging in size from 506 to 829,175 bp. The assembled genome had 121-fold coverage, with an *N*_50_ scaffold size of 183,112 bp.

The draft genome sequence of *P. chlororaphis* EA105 consists of 6,595,581 bp, with a G+C content of 59.2%. Annotation was performed using the NCBI PGAP pipeline, which predicted 5,655 protein-coding sequences and 53 tRNAs. The 5S, 16S, and 23S rRNA genes were detected, but because the assembly is based on short reads, the numbers and locations of multiple copies could not be determined. An initial analysis of the genome sequence for genes related to antifungal activity and interactions with other species revealed a cluster of genes putatively involved in synthesis of hydrogen cyanide (HCN) in contig 20 (11), but unlike other *P. chlororaphis* strains (2, 6, 8, 12, 13), we did not find genes specifying pyrrolnitrin or phenazine (14, 15), nor did we find evidence of the ability to produce two other well-known biocontrol metabolites pyoluteorin or 2,4-DAPG (16, 17). Clusters of genes associated with type VI secretion structural components were found on contigs 2 and 24, similar to *Pseudomonas aeruginosa* PAO1’s locus 1-type and locus 4A-type, respectively (18). We also found numerous hits to putative type VI secretion system effectors from a variety of *Pseudomonas* species, including some clustered with the structural components in contigs 2 and 24. This genome sequence will enable further comparative genomic studies among *P. chlororaphis* strains and facilitate genetic and transcriptomic analysis of the plant protection capability of *P. chlororaphis* EA105.

### Nucleotide sequence accession numbers.

This whole-genome shotgun project has been deposited at DDBJ/EMBL/GenBank under the accession no. JSFK00000000. The version described in this paper is version JSFK01000000.
